# Inhalation of Ether Previous to Surgical Operations

**Published:** 1847-04

**Authors:** 


*Inhalation of Ether previous to Surgical Operations.* By J. Mason Warren, M. D., one of the Surgeons of the Massachusetts General Hospital.

Dr. Warren reports for the Boston Medical and Surgical Journal nineteen cases, in which, at the Massachusetts General Hospital, and in private practice, the Ethereal vapor was successfully administered previous to surgical operations. He deduces from the results in these cases the following conclusions: — Editor.

A. *As to age and temperament.*—1st. Young Children and females seem most easily to be brought under its influence.

2nd. Females of nervous temperament are not unfrequently brought to a condition closely resembling hysteria.

3rd. Men of powerful muscular frames sometimes become violently frantic at first, requiring the exertions of several persons to restrain them. This state is succeeded by one of semi-consciousness, but also of insensibility to pain.

B. *As to the method to be employed.*—1st. The use of the ordinary inhaling apparatus seemed in many cases to occasion at first irritation and choking.

2nd. This irritation either does not exist, or in a less degree, in cases where cloth or sponge has been used, which has been pretty extensively employed at the Hospital and in private practice by Dr. J. C. Warren and myself.

3rd. In many cases it is impossible, in consequence of the tender age of the patient, or his refractory nature, to make him comprehend the use of the ordinary apparatus, and here the cloth or sponge will be found of great service.

4th. A quieting effect is produced even when ether is sprinkled upon the bed clothes, or a sponge moistened with it is laid upon the pillow ; thus sometimes superseding the use of an opiate.

C. *As to its influence and effect.*—1st. When the effect is perfect, the patient having recovered, generally expresses himself as previously under the influence of a pleasant dream, notwithstanding the severity of the operation.

2nd. A partial effect is often produced, in which the patient is entirely free from pain, but yet conscious of the different steps of the operation. Uneasiness, apparently the result of suffering, is in these instances generally referred to a disagreeable dream, or to some cause not immediately connected with the operation.

3rd. In some cases asphyxia is produced, requiring the admission of fresh air, with the use of frictions, to the patient, and in the most severe cases. the internal administration of stimulants.

4th. Repetition increases the susceptibility, so far as observed.

5th. No limit of time can as yet be assigned at which its use becomes unsafe. In one instance here recorded the patient was kept under its influence for a half, and in another for three quarters of an hour.

6th. The effect is generally evanescent, and the patient being spared the shock of severe pain upon his system seems to recover more rapidly than in ordinary cases. Out of more than fifty instances, which have fallen under my observation, I am not aware of a single one, in which its use has proved permanently deleterious.

7th. Various observations show, that when the patient is to undergo a severe operation under the influence of the ether, its application should be employed repeatedly before the day of the operation, as well to instruct him how to respire it thoroughly, as to ascertain the peculiar manner in which it affects him.

